# Proteolipid Protein 2 Overexpression Indicates Aggressive Tumor Behavior and Adverse Prognosis in Human Gliomas

**DOI:** 10.3390/ijms19113353

**Published:** 2018-10-26

**Authors:** Yi-Hsuan Chen, Dueng-Yuan Hueng, Wen-Chiuan Tsai

**Affiliations:** 1 Graduate Institute of Pathology and Parasitology, National Defense Medical Center, Taipei 11490, Taiwan; ab95057@hotmail.com; 2 Department of Neurological Surgery, Tri-Service General Hospital, National Defense Medical Center, Taipei 11490, Taiwan; hondy2195@yahoo.com.tw; 3 Department of Pathology, Tri-Service General Hospital, National Defense Medical Center, No. 325, Sec. 2, Cheng-Kung Road, Neihu 114, Taipei 11490, Taiwan

**Keywords:** PLP2, glioma, survival time, WHO grade

## Abstract

Proteolipid protein 2 (PLP2), a membrane protein of the endoplasmic reticulum, is related to tumor proliferation and metastasis in some human cancers, but not in gliomas. First, we performed western-blot analysis, real-time quantitative PCR and immunohistochemical stains to detect PLP2 expression in 4 glioma cell lines and human glioma tissues. In addition, we used small interfering RNA (SiPLP2) and short hairpin RNA (shPLP2) to knockdown PLP2 expression in GBM8401 and LN229 glioma cell lines. After then, the alteration of PLP2 suppressed glioma cells behavior were examined by cell proliferation, wound healing, cell invasion, and colonies formation assays. Finally, the possible mechanism of PLP2 was analyzed by detecting the expression of the proteins related to cell-cycle checkpoints, cell-proliferative signaling factors, and cell-matrix interaction. Compared with normal brain cell lysates and mRNA, all glioma cell lines displayed PLP2 protein and mRNA overexpression. Besides, higher PLP2 IHC staining significantly correlated with more advanced tumor grades and poorer prognosis in human gliomas. Both siPLP2 transfected gliomas showed a clear inhibition of glioma cell proliferation, migration, and invasion as well as down-regulating p-p38, p-ERK, MMP-2, and MMP-9 expression. In conclusion, we successfully demonstrated that PLP2 overexpression played an oncogenic role in glioma development and aggressive tumor behavior.

## 1. Introduction

Primary brain tumors (PBT) are not easily-identified neoplasms because of the limitations of diagnostic tools and the inconspicuous symptoms, except for tumor compression [1]. Whether PBTs are benign or malignant, surgical resection of brain neoplasms is a standard treatment to decrease tumor recurrence rates and prolong patient survival [2,3]. In a recent study, various molecular epidemiologic factors, including unrepaired mutant DNA, cell-cycle disarrangement, metabolic disorders, and long-term repeated inflammation, have been demonstrated to be related to the development of PBTs [4]. The two most common categories of PBTs are meningiomas and gliomas [5,6]. Optimal treatment and prognosis depend on the extent of tumor involvement, tumor size, location, and World Health Organization (WHO) specified grades [7]. Unlike low-grade glioma, post-operative radiotherapy and chemotherapy produced some benefits with more advanced tumors [7]. In recent years, the concomitance of newly developed alkylating drugs, such as temozolomide (TMZ), with external beam radiation therapy or stereotactic radiosurgery has been shown to effectively induce apoptosis in high-grade glioma cells and potentially result in prognostic improvement. This concomitance is especially apparent in elderly patients [7,8]. However, some severe complications from the above therapeutic regimens, including somnolence syndrome [9], cerebral radionecrosis [10], posterior leukoencephalopathy [11,12], and secondary hematologic malignancies [13,14], could restrict the wide use of these drugs. Recently, many genetic mutations have been proven to be related to drug effectiveness and the disease-free survival of glioma patients. Such mutations include those of O^6^ methyl-guanine methyltransferase (*MGMT*) [15,16], isocitrate dehydrogenase (*IDH*) [17,18], *BRAF* [19,20], and co-deletion of 1p and 19q [21,22,23]. Similarly, some genetic aberrations, such as in NF2 [24,25], *AKT1/TRF7* [26], *SMO* [26], *KLF4/TRF7* [27], and *TERT* [28], have been demonstrated to be associated with the tumor recurrence rate, histological sub-classification, and disease-free survival time of meningioma patients. Accordingly, PBTs are considered a multifactorial disease [5].

According to the revised 2016 WHO classification of central nervous system tumors, grade II to IV astrocytic tumors divided into IDH-mutant and IDH-wildtype based on the immunohistochemical analysis. The function of IDH catalyzes the oxidative decarboxylation of isocitrate, which produces alpha-ketoglutarate [29]. The mutation status of IDH1 or IDH2 leads to the production of the oncometabolite 2-hydroxyglutarate [29]. The epidemiology of IDH mutation mainly located on grade II–III gliomas and represented a relatively favorable prognosis [4]. However, only a small portion of glioblastomas revealed IDH mutation. In addition, compared to other high-grade gliomas, a new entity of “diffuse midline glioma, H3 K27M-mutant” often occurred in children [30]. The mutation of histone H3 often located on at codon 27 and represented a gain of function [31]. H3 K27M mutation gliomas showed aggressive tumor behavior and poor prognosis, even histological absence of brick mitotic figures, microvascular proliferation, or pseudopalisading necrosis [32].

The phosphatidylinositol-3-kinase (PI3K)/protein kinase B (Akt) and the mammalian target of rapamycin (mTOR) signaling pathways induce cell proliferation and angiogenesis in glioblastomas and neuroblastomas [33,34]. The suppression of PI3K/AKT/mTOR pathway effectively regulates cell-cycle entry, glycogen metabolism, and vasculogenesis [35]. Proteolipid protein 2 (PLP2) is a 4-transmembrane protein that is expressed in several areas of the brain, including the hippocampus [36]. Otherwise, PLP2 had been viewed as an oncogenic-inducer in several human cancers including melanoma, osteosarcoma, breast cancer, hepatocellular carcinomas, and acute lymphoblastic leukemia [37,38,39,40,41]. In the recent study, PLP2 could stimulate matrix metalloprotease 2 (MMP2) secretions to induce melanoma cell proliferation, invasion and even metastasis [37]. However, the function of PLP2 in gliomas remained unclear.

In this study, we performed in vitro studies, tissue microarrays, and immunohistochemical stains to detect the possible role of PLP2 in glioma. This study successfully proves that PLP2 induces tumor overgrowth and correlates with poor prognosis in glioma patients. Additionally, PLP2 suppression may inhibit glioma cell migration and invasion. Although the detailed mechanism remained undetermined, our results supported PLP2 could induce cell cycle checkpoint dysregulation, stimulate extracellular matrix factors overexpression and enhance Raf/MEK/ERK signaling pathway in glioma tumorigenesis. Furthermore, the consistent results from in vitro studies and human tissue specimens supplied strong evidence to prove the oncogenic role of PLP2 in glioma.

## 2. Results

### 2.1. PLP2 Protein Overexpression in Human Glioma Cell Lines

**Figure 1:**
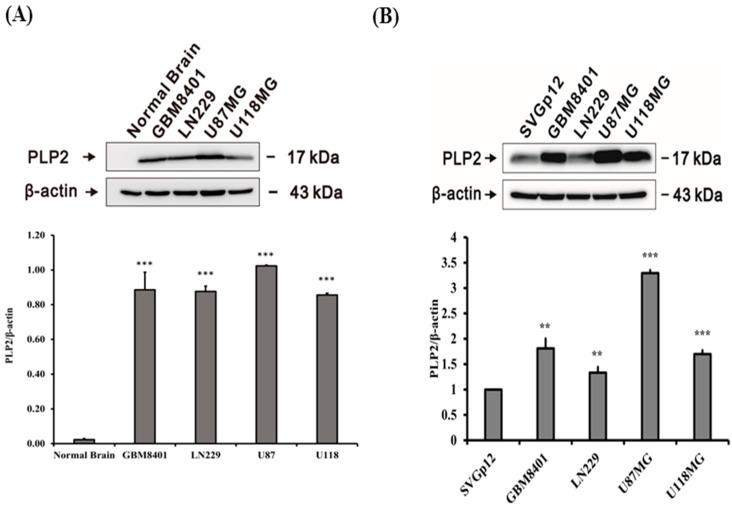
Expression analysis of proteolipid protein 2 (PLP2) in glioma cell lines and normal brain tissues. (**A**) Analysis of PLP2 protein expression in GBM8401, LN229, U87, and U118MG glioma cell lines and normal brain tissue protein lysates. (**B**) Analysis of PLP2 protein expression in GBM8401, LN229, U87, and U118MG glioma and the human fetal glial cell line SVG p12 glial cell lines. The densitometric analysis revealed a higher percentage of peak of PLP2 (17 kDA) in all glioma cell lines than in normal brain tissue protein lysates and SVG p12 glial cell line. (** *p* < 0.01; *** *p* < 0.001).

To detect PLP2 protein expression, western-blot analysis was performed in normal brain tissue and human glioma cell lines. Compared with normal brain cell lysates, our study revealed PLP2 overexpression in the GBM8401, LN229, U87MG, and U118MG human glioma cell lines (* *p* < 0.05; ** *p* < 0.01; *** *p* < 0.001, Figure 1A). In order to evaluate the differences of PLP2 expression between glial cell and glioma cell lines, higher PLP2 expression was identified on all glioma cell lines than the SV40-immortalized human fetal glial cell line SVG p12 by western-blot analysis (** *p* < 0.01; *** *p* < 0.001, Figure 1B). Therefore, in an in vitro study, we demonstrated the phenomenon of PLP2 overexpression in all human glioma cell lines.

### 2.2. Higher PLP2 mRNA Expression in Human Glioma Cell Lines than in Normal Brain Tissues

**Figure 2:**
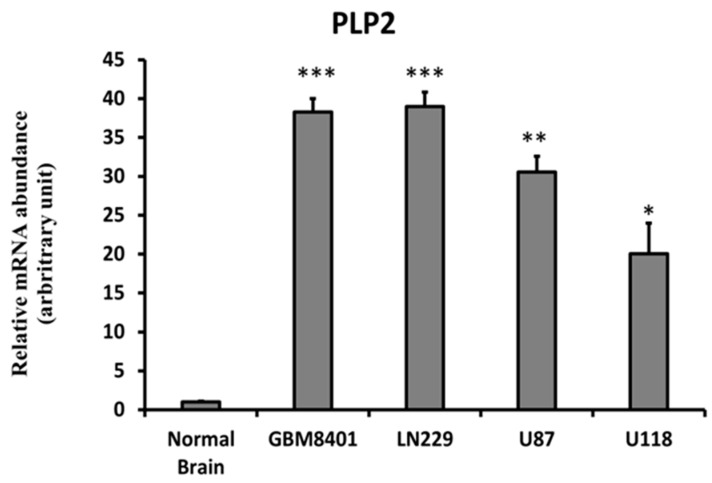
Using quantitative RT-PCR to detect PLP2 mRNA expression in GBM8401, LN229, U87, and U118MG glioma cell lines and normal brain tissue cDNA.β-actin served as a loading control. Data are representative of three independent experiments. (*p < 0.05; **p < 0.01; *** *p* < 0.001).

To evaluate PLP2 mRNA expression in human glioma cell lines, we applied quantitative RT-PCR on cDNA isolated from normal brain tissue and four different clones of glioma cell lines including GBM8401, LN229, U87MG, and U118MG. All glioma cell lines presented higher PLP2 mRNA expression than normal brain tissue cDNA. Of all glioma cell lines, the degree of PLP2 mRNA expression in LN229 and GBM8401 was higher than that in U87MG and U118MG (** *p* < 0.01; *** *p* < 0.001, Figure 2).

### 2.3. PLP2 IHC Staining Correlated with WHO Grades and Poor Prognosis in Gliomas

**Figure 3:**
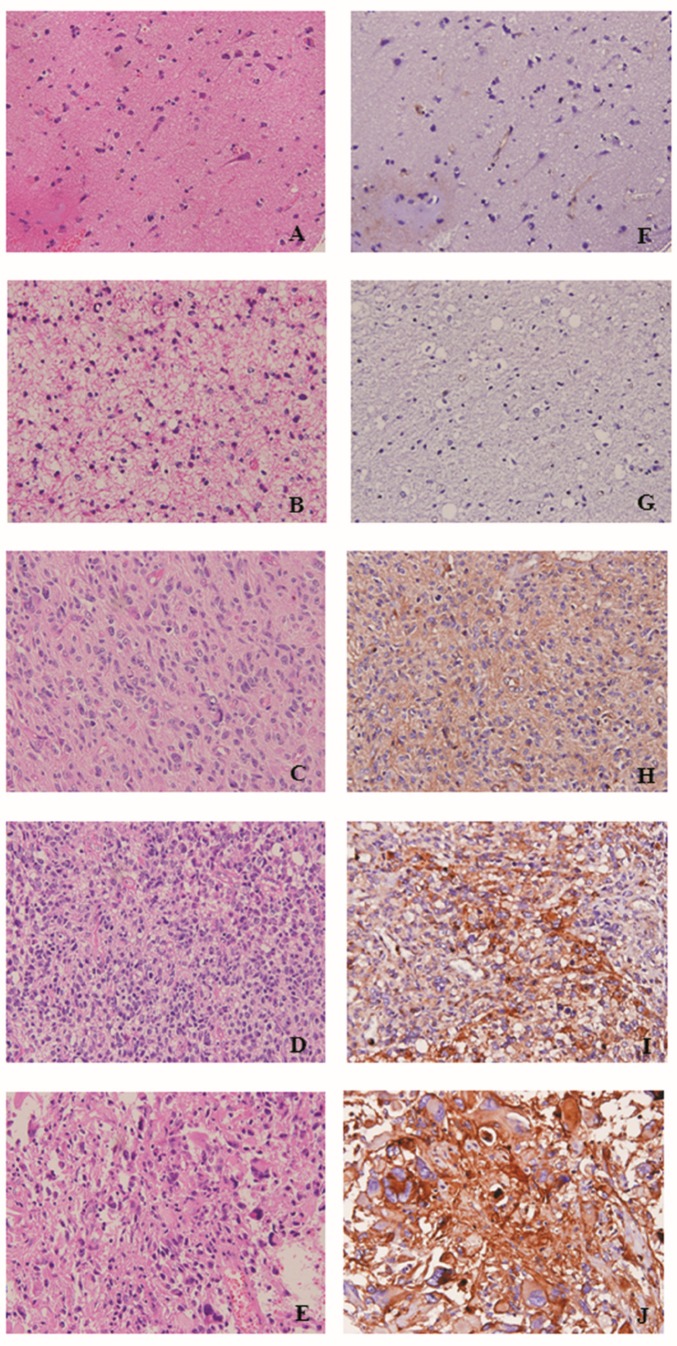
Hematoxylin and eosin staining of (**A**) normal brain tissue, (**B**) pilocytic astrocytoma, (**C**) diffuse astrocytoma, (**D**) anaplastic astrocytoma, and (**E**) glioblastome multiforme. IHC analysis of PLP2 in (**F**) normal brain tissue, (**G**) pilocytic astrocytoma, (**H**) diffuse astrocytoma, (**I**) anaplastic astrocytoma, and (**J**) glioblastoma. (Original magnification × 400).

**Figure 4:**
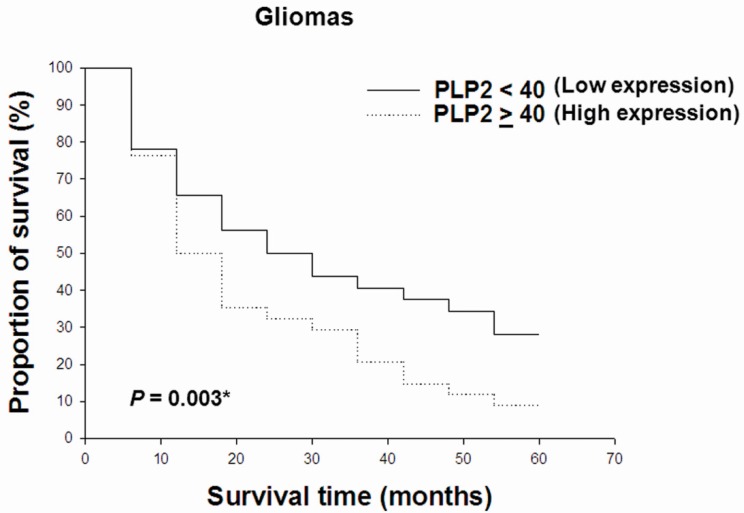
Relationships between overall survival rate and PLP2 immunostain scores in gliomas. Survival rates were analyzed using the Kaplan–Meier survival test (* *p* < 0.05).

To evaluate PLP2 expression in human glioma tissues, we performed IHC staining for PLP2 on 2 tissue microarray slides of various World Health Organization (WHO) grades of gliomas. After the exclusion of some cases with inadequate representative specimens, 10 non-neoplastic brain tissues and 76 gliomas with various WHO grades were included in this study. Of all gliomas, 1 pilocytic astrocytoma, 1 diffuse astrocytoma with IDH-mutant, 13 diffuse astrocytoma with IDH-wildtype (WT), 3 anaplastic astrocytomas with IDH-mutant, 7 anaplastic astrocytomas with IDH-WT, 3 oligodendrogliomas, NOS, 5 anaplastic oligodendrogliomas, NOS, 5 glioblastomas with IDH-mutant, 26 glioblastomas with IDH-WT, and 12 diffuse midline gliomas with H3 K27M-mutant from the results of IDH1 R132H, IDH2 and H3 K27M IHC staining (Table 1). Most non-neoplastic brain tissue cases revealed negative or small portions of PLP2 IHC staining. On the contrary, higher PLP2 expression significantly correlated with more advanced tumor grades of gliomas (*p* = 0.039, Figure 3). Otherwise, in order to evaluate PLP2 with other glioma-related genes expression, we performed univariate or multivariate analysis to detect the possible PLP2 associated risk factors. The epidermal growth factor receptor vIII (EGFRvIII), AXL and loss of H3 K27me3 expression were the independent factors associated with PLP2 overexpression (Table 2). Additionally, to detect the overall survival time with PLP2 expression, we divided all gliomas into two groups based on high and low PLP2 expression. The cut-off of PLP2 immunostain scores was set at 40 for gliomas based on the relatively equal number of cases in each group [42]. 32 cases of PLP2 immunostain score < 40 and 34 cases of score ≥ 40 were followed for five years. These data revealed that higher PLP2 expression was correlated with shorter overall survival time in gliomas (*p* = 0.003, Figure 4). Furthermore, of all included factors, senior glioma patient, PLP2 overexpression, loss of ATRX, and positive expression of AxL, NUR77 or PDGFRA were the independent prognostic factors under cox regression analysis (Table 3).

### 2.4. Suppression of PLP2 Inhibits Glioma Cell Proliferation

**Figure 5:**
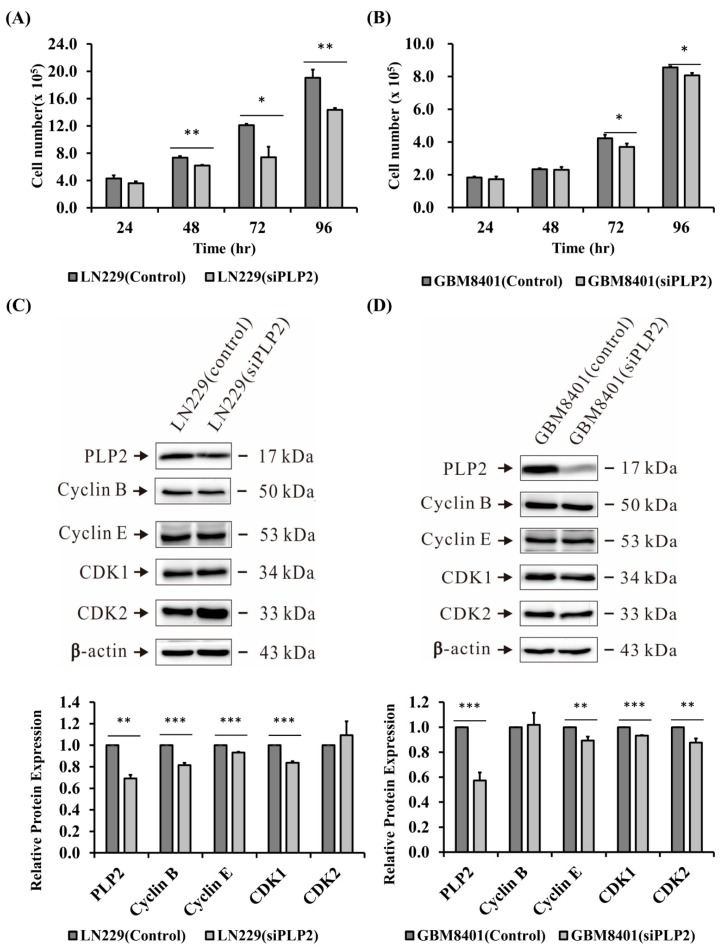
Analysis of (**A**) LN229 and (**B**) GBM8401 glioma cells proliferative ability after using small interfering RNA (siPLP2) to knockdown PLP2 expression. (* *p* < 0.05; ** *p* < 0.01); Using western-blot analysis to detect the cell-cycle checkpoints expression in (**C**) LN229 and (**D**) GBM8401 glioma cell lines after siPLP2 administration (** *p* < 0.01; *** *p* < 0.001).

**Figure 6:**
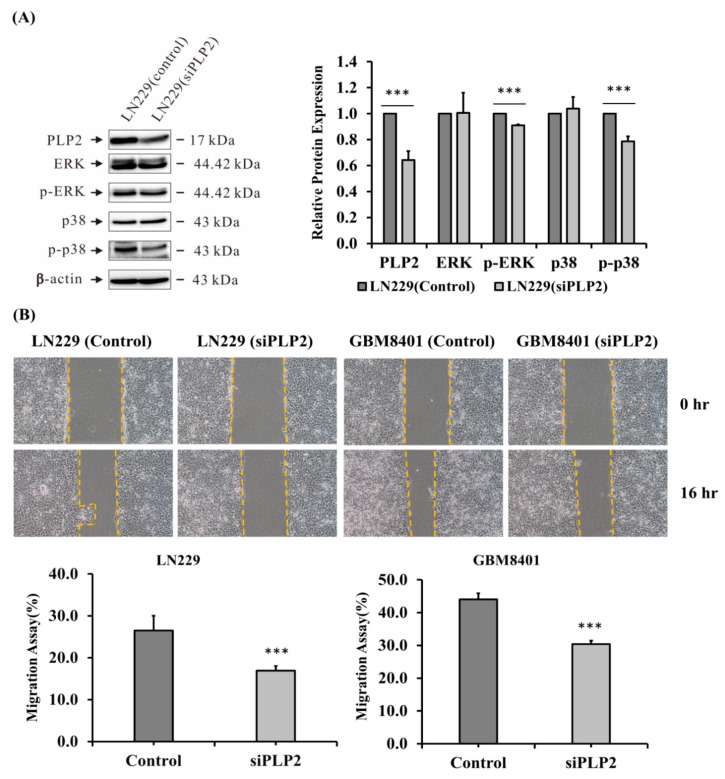
(**A**) Influence of the possible signaling pathway in the LN229 glioma cell line after siPLP2 and siRNA-A (NC)transfection. p-p38 and p-ERK showed a significant decrease after knockdown PLP2 expression. (*** *p* < 0.001); (**B**) Assessment of tumor migration ability in GBM8401 and LN229 glioma cell lines after siPLP2 and NC transfection. Glioma cells with a PLP2 knockdown revealed slow tumor migration after 16 h. (*** *p* < 0.001).

To check the role of PLP2 expression and glioma cell proliferation, cell counting assessment was analyzed. Since GBM8401 and LN229 glioma cell lines revealed higher PLP2 mRNA expression than U87MG, and U118MG, GBM8401 and LN229 glioma cell were transfected with siPLP2 and siRNA-A (scramble siRNA) (NC). In the first 24 h, the number of siPLP2 transfected glioma cells was not significantly different from that in the NC transfected group. After 48 h, siPLP2 transfected LN229 glioma cells revealed lower ability for cell proliferation (* *p* < 0.05; ** *p* < 0.01, Figure 5A). Similarly, compared with NC, a significantly lower number of GBM8401 glioma, with siPLP2 transfection, was also identified after 72 h (*p* < 0.05, Figure 5B). Therefore, we successfully demonstrated that PLP2 is an important cell proliferative factor in glioma cells, especially the LN229 glioma cell line. Additionally, we also performed western-blot analysis to evaluate the association between PLP2 and cell-cycle checkpoints in the LN229 and GBM8401 glioma cells. Our study revealed that the inhibition of PLP2 could suppress cyclin-B, cyclin-E, and CDK1 expression in LN229 glioma cells (** *p* < 0.01; *** *p* < 0.001, Figure 5C). In addition, cyclin-E, CDK1, and CDK2 were significantly suppressed in siPLP2 transfected GBM8401 gliomas (** *p* < 0.01; *** *p* < 0.001, Figure 5D). Therefore, we confirmed that PLP2 could accelerate cell proliferation depending on the activation of cell-cycle checkpoint proteins from the G1-S and G2-M phases. Furthermore, our data also revealed PLP2 inhibition could effectively down-regulate phosphorylated-p38 (p-p38) and p-ERK expression (*p* < 0.001, Figure 6A).

### 2.5. PLP2 Inhibition Down-Regulates Glioma Cell Migration and Invasion

**Figure 7:**
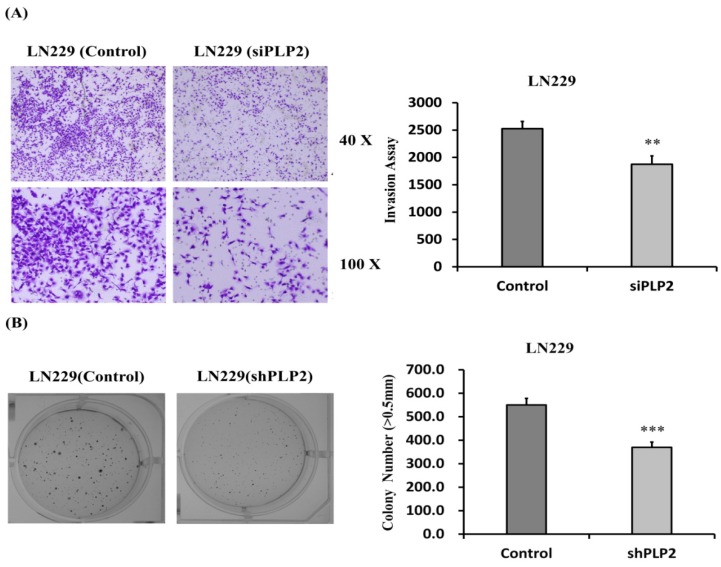
(**A**) The cell invasive ability in LN229 glioma cell line with siPLP2 transfection was lower than NC transfection after 24 h incubation; (**B**) Similarly, siPLP2 transfected LN229 glioma cells revealed lesser cell colonies then NC transfected tumors. (** *p* < 0.01; *** *p* < 0.001).

To detect the relationship between PLP2 expression and glioma cell aggressiveness, wound-healing tests, and cell invasion assays were employed on GBM8401 and LN229 cell lines. Both the included glioma cell lines with siPLP2 transfection showed weaker cellular migration than NC transfection after 16 h as determined by the difference in the dimensions of the scratch area (*p* < 0.001, Figure 6B). Similarly, less trans-membranous siPLP2 transfected LN229 glioma cells indicated that the suppression of PLP2 could inhibit tumor invasive behavior (*p* < 0.01, Figure 7A).

### 2.6. Knockdown of PLP2 Expression Inhibits Glioma Tumorigeneity

**Figure 8:**
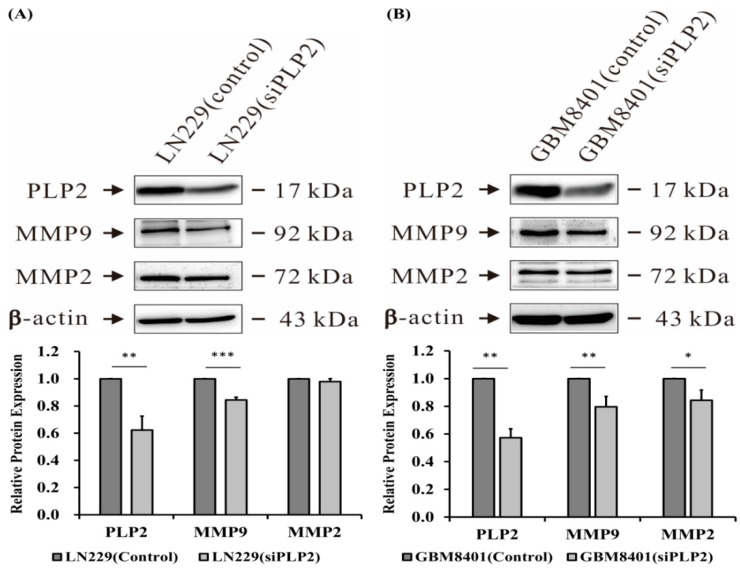
The detection of tumor cells and matrix interaction analyzed by metalloproteinase 2 (MMP2) and metalloproteinase 9 (MMP9) expression between the groups of siPLP2 and NC transfected (**A**) LN229 and (**B**) GBM8401 glioma cells. Compared to NC transfection, both of above glioma cells with siPLP2 transfection revealed the inhibition of MMP9 expression. In addition, the suppression of MMP2 by down-regulation of PLP2 is identified in GBM8401 glioma cell line, but not in LN229. The densitometric analysis revealed a lower percentage of peaks on MMP2 (72 kDA) and MMP9 (92 kDA) in LN229 (siPLP2) than in LN229 (control). Similarly, lower percentage of peak on MMP9 (92 kDA) by densitometric analysis is identified in GBM8401 (siPLP2) than in GBM8401 (control) (* *p* < 0.05; ** *p* < 0.01; *** *p* < 0.001).

Since soft agar colony formation test is an ideal tool for quantitative assessing cancer cell proliferation and migration [43], we performed this assay to evaluate the ability for tumor tumorigenicity in gliomas with a PLP2 knockdown. Our results indicate that the LN229 glioma cells with short hairpin RNA (shPLP2) transfection produce significantly fewer cell colonies than cells transfected with an empty-vector (*p* < 0.001, Figure 7B). Therefore, the inhibition of PLP2 expression could effectively decrease glioma cell spheres formation. Besides, as the extracellular matrix degradation is an important factor for tumor invasion and metastasis, we also evaluated the possible influence of PLP2 expression and cell-matrix interaction. These data indicate that PLP2 knockdown could down-regulate matrix metalloproteinase 9 (MMP9) in LN229 and GBM8401 glioma cell lines, and matrix metalloproteinase 2 (MMP2) expression in GBM8401 glioma cell line (* *p* < 0.05; ** *p* < 0.01; *** *p* < 0.001, Figure 8).

## 3. Discussion

Recently, PLP2 expression has been reported to be associated with tumor cell persistence and metastasis in melanoma and hematologic malignancies [37,41]. Sonoda et al. [37] demonstrated that PLP2 plays an important role in up-regulation of the PI3K/AKT/mTOR pathway and induction of melanoma cell overgrowth. Additionally, the down-regulation of PLP2 expression by microRNA-664 significantly inhibited leukemic and melanoma cell proliferation and invasion [41,44]. Similarly, we demonstrated the relationship between PLP2 expression and glioma cell proliferation, migration, invasion, and metastatic characters. We also identified the potential activation of G2-M cell-cycle checkpoints, and factors involved in cell-matrix interaction, as possible mechanisms in the in vitro studies. In addition, our tissue microarray study showed that PLP2 IHC staining is positively correlated with tumor grade and poor survival time in human gliomas.

In the current study, our results indicated the possible role of PLP2 in the up-regulation of the phosphorylated p38 (p-p38) and p-ERK. Bradham et al. [45] demonstrated that p38 is a mitogen-activated protein kinase (MAPK) which acts as a responder to stress and cytokines. p38 activation is associated with several signaling pathways, such as MAPK and MKK kinase [46]. In addition, p38, MAPK, phosphatidylinositol 3-kinase (PI3K), Rac1, Cdc42, and JNK-dependent pathways are important factors for lysophosphatidic acid (LPA) signaling, which is related to glioma cell migration [45]. Otherwise, ERK phosphorylation relied on some cellular proliferative factors, such as guanine nucleotide binding protein (G-protein) coupled receptors and carcinogens [47]. The processes of cellular mitosis and meiosis were involved in the Raf/MEK/ERK signaling pathway [48]. Therefore, we discovered that PLP2 expression could up-regulate the p-p38 and p-ERK pathways and induced glioma cell proliferation, migration, and invasion. However, more evidence is needed to confirm the detailed mechanism.

It is difficult to discriminate reactive glial proliferation from low-grade neoplastic lesions because of the overlap in histologic appearance and the similar proliferative index [49,50]. Moreover, it is difficult to determine the precise glioma WHO grades owing to the frequently fragmented surgical specimens taken from limited operative spaces. In the recent studies, the benefits of IHC stain analysis for the assessment of glioma cell behavior was shown to be relatively effective and inexpensive [51,52]. The application of isocitrate dehydrogenase I R132H (IDH1 R132H) could differentiate low-grade gliomas from non-neoplastic CNS lesions [53,54]. In this study, we showed that PLP2 IHC staining can not only discriminate glioma from non-neoplastic brain tissue, but also help clinical doctors and pathologists predict tumor aggressiveness and overall prognosis.

From our univariate and multivariate analysis, PLP2 overexpression was associated with the activation of some oncogenic factors or the inhibition of tumor suppressor genes, including H3 K27M, MGMT, EGFRvIII, AxL, p-AxL, NUR77, and H3 Lys27. EGFRvIII is an autophosphorylated-form of EGFR and presents in half of glioblastomas with EGFR amplification [55]. Overexpression of EGFRvIII is viewed as a poor prognostic factor because of its role on tumor invasion and metastasis as well as angiogenesis [56]. Besides, AxL is the tyrosine kinase receptor and plays a role as an adhesion molecule of immunoglobulin [57]. The combination of AxL and growth arrest-specific gene 6 (Gas6) is responsible for tumor progression and migration [58]. Otherwise, the orphan receptor NUR77 is a transcription factor and regulates T cell apoptosis [59]. In the recent study, the inhibition of NUR77expression could induce glioma cell survival via down-regulating JNK pathway [60]. In addition, the results of cox regression analysis revealed PDGFRA and above oncologic factors are independent prognostic factors in gliomas. Recently, Kessler et al. have demonstrated the amplification of platelet-derived growth factor receptor alpha (PDGFRA) related to glioblastoma progression, especially in the methylated form of MGMT tumors [61]. Therefore, in this study, we firstly demonstrated the upregulation of PLP2 expression could be possibly related to *AxL*, *p-AxL*, *EGFRvIII*, *PDGFRA*, *NUR77*, *H3 K27M*, *H3 Lys27*, and *MGMT* genetic mutation, but the detailed mechanism needed more evidence.

## 4. Materials and Methods

### 4.1. Human Glioma Cell Lines and Lysate Preparation

Human glioma cell lines GBM8401, LN229, U87MG, and U118MG were purchased from the Cell Resource Center, Shanghai Institute of Biochemistry and Cell Biology at the Chinese Academy of Sciences (Shanghai, China). GBM8401, LN229, U87MG, and U118MG were maintained in Dulbecco’s modified Eagle’s medium (DMEM) containing 13.1 mM NaHCO3, 13 mM glucose, 2 mM glutamine, 10% fetal bovine serum (FBS), 100 U/mL penicillin, and 100 mg/mL streptomycin. Cultures were maintained in a humidified incubator with 5% CO_2_ at 37 °C. At the end of incubation, the cells were lysed by adding lysis buffer containing 10 mM Tris-HCl, 1 mM EGTA, 1 mM MgCl2, 1 mM sodium orthovanadate, 1 mM DTT, 0.1% mercaptoethanol, 0.5% Triton X-100, and protease inhibitor cocktails. Cell lysates were prepared from 2 × 10^7^ cells from the GBM8401, LN229, U87MG, and U118MG glioma cell lines. Lysate was used for Western blot analysis for PLP2, with β-actin as an internal control. In addition, normal brain cell lysates prepared from cerebral cortex were purchased from the company of Biocompare (MBS537208, San Francisco, CA, USA).

### 4.2. Western Blot Analysis

Polyclonal rabbit anti-human PLP2 antibody (1:5000, ab180131, Abcam, Cambridge, UK), and monoclonal mouse anti-β-actin antibody (1:5000, sc47778, San, St. Louista Cruz, TX, USA) were employed as primary antibodies in western blot analysis. The analysis was performed following the previously described protocol [38].

### 4.3. RNA Isolation and Real-Time Reverse Transcription-PCR

Total RNA was extracted (PAXGeneTM Blood RNA kit, PreAnalytix, Hombrechtikon, Switzerland) and residual genomic DNA was digested using the RNase-Free DNase set (Qiagen, Hilden, Germany). Single-stranded cDNA was prepared from 1 µg of total RNA using the Thermoscript RT-PCR system (Invitrogen, Carlsbad, CA, USA). The mRNA expression of selected genes was quantified using qRT-PCR. PCR reactions performed with a LightCyclerTM instrument using the Fast-StartTM DNA Master SYBR Green I real-time PCR kit (Roche Molecular Biochemicals, Mannheim, Germany). Thermocycling was performed with a final volume of 20 µL of 3 mM magnesium chloride (MgCl_2_), 0.5 µM each of the required primers, and 10 µL of the appropriate cDNA dilution. PCR was performed with an initial denaturation step of 10 min at 95 °C, followed by 40 cycles of a touch-down PCR protocol (10 s at 95 °C, 10 s annealing at 68–58 °C, and 16 s extension at 72 °C). Specific primers for GAPDH and PLP2 (list as follows) were purchased from Search-LC (Heidelberg, Germany). To confirm amplification specificity, the PCR products from each primer pair were subjected to a 58–98 °C temperature 0.1 °C/s) for melting curve analysis.

GAPDH: 5′-GCACCGTCAAGGCTGAGAAC-3′ (forward)

GAPDH: 5′-ATGGTGGTGAAGACGCCAGT-3′ (reverse)

PLP2: 5′-CTCATAGCGGCAATCCTCTACC-3′ (forward)

PLP2: 5′-CTGCGACGATTTTGGAGTGG-3′ (reverse)

### 4.4. Tissue Microarray Slide Preparation and Immunohistochemistry

Tissue microarray slides (No. GL2083a and GL2083b) were purchased from GenDiscovey Biotechnology Inc. According to the 2016 World Health Organization Classification of Tumors of the Central Nervous System [4], we performed some biomarkers to re-classify all included gliomas and detect the possibly PLP2-related factors. Initially, the Antigen retrieval was performed by heating (100 °C) each section for 30 min in a 0.01 mol/L sodium citrate buffer (pH 6.0). After 3 rinses (each for 5 min in phosphate buffered saline [PBS]), sections were incubated for 1 h at room temperature with a polyclonal rabbit anti-human PLP2 antibody (1:100, Biorbyt, Cambridge, UK), a monoclonal mouse anti-human IDH1 R132H antibody (1:100, Dianova, Hamburg, Germany), a polyclonal rabbit anti-human ATRX antibody (1:100, ATLAS, Stockholm, Sweden), a polyclonal rabbit anti-human Histone H3, K27M mutant (H3 K27M) antibody (1:100, Millipore, Bedford, CA, USA), a polyclonal rabbit anti-human Trimethyl-Histone H3, Lys27 (H3 K27me3) antibody (1:1000, Millipore, Bedford, CA, USA), a monoclonal mouse anti-human EGFR antibody (1:100, Thermo Fisher Scientific, San Jose, CA, USA), a monoclonal mouse anti-human EGFRvIII antibody (1:100, Absolute, Oxiford, UK), a polyclonal rabbit anti-human AXL antibody (1:50, Sigma-Aldrich, St Louis, MO, USA), a monoclonal mouse anti-human phosphor-Axl (Y779) antibody (1:50, R&D system, Minneapolis, MN, USA), a monoclonal mouse anti-human p53 antibody (1:100, DAKO, Carpinteria, CA, USA), a monoclonal mouse anti-human Neurofilament antibody (1:100, DAKO, Carpinteria, CA, USA), a monoclonal mouse anti-human platelet-derived growth factor-alpha (PDGFRA) antibody (1:100, Santa Cruz Biotechnology, Santa Cruz, TX, USA), a polyclonal rabbit anti-human neurofibromin (NF1) antibody (1:100, Abcam, Cambridge, UK), a polyclonal rabbit anti-human NUR77 antibody (1:100, Abcam, Cambridge, UK) diluted in PBS. After 3 washes (each for 5 min in PBS), the sections were incubated with biotin-labeled secondary immunoglobulin (1:100, DAKO, Glostrup, Denmark) for 1 h at room temperature. After 3 additional washes, peroxidase activity was developed with a 3-amino-9-ethylcarbazole substrate chromogen (DAKO, Glostrup, Denmark) at room temperature.

### 4.5. Assessment of Immunostain Scores in Gliomas

To assess PLP2 expression, all tumor cells with membranous and cytoplasmic staining were assessed. The intensity of cytoplasmic and membranous immunostain of tumor cells was scored on a scale of 0 (absence of staining), 1 (weak staining), 2 (moderate staining), or 3 (strong staining). Weak, moderate, and strong cytoplasmic staining were identified based on microscopy at a magnification of 40×, 20×, and 10× or 4× respectively. In addition, the percentage of tumor cells with cytoplasmic or nuclear staining was estimated. The percentage of cells (from 0 to 100) at each intensity was multiplied by the corresponding immunostaining intensity (from 0 to 3) to obtain an immunostaining score ranging from 0 to 300. For biomarker analysis, tumors with <5% cytoplasmic and nuclear staining were considered negatively stained. The immunohistochemical stains for PLP2 in normal colonic mucosa were selected as positive controls. Otherwise, the criteria of positive results of the other biomarkers except PLP2 depended on equal or more than 5% of tumor expression.

### 4.6. Statistical Analysis of PLP2 Expression

To detect the correlation of PLP2 IHC stain with WHO grades and glioma survival rates, statistical analysis was performed using the Pearson Product Moment method and Kaplan-Meier survival test. With a *p* value less than 0.05, correlation of tumor grades with PLP2 immunoscores was established. In addition, survival times were calculated from the date of surgery to the date of death. These cases are divided into 2 groups to compare the survival time with the PLP2 immunostain scores. Statistical analysis of survival time was done with the Kaplan-Meier survival test. Furthermore, we also performed univariate and multivariate analyses and cox proportional hazards regression to evaluate the possible independent factors related to PLP2 overexpression and prognosis.

### 4.7. Transfection of siPLP2 and siRNA-A into LN229 and GBM8401 Cell Lines and Cell Proliferation Assay

For transfection studies, PLP2 siRNA (siPLP2) and siRNA-A were purchased from Ambion (assay ID, PM10206 and AM10206). The role of siRNA-A was used as the negative control (NC). LN229 and GBM8401 cells were transfected with 100 µM siPLP2 and NC in the 6 cm dish using Lipofectamine 2000 transfection reagent (Invitrogen, Carlsbad, CA, USA). Cells from each group were seeded at 5 × 10^4^ in 12 well plates and incubated overnight at 37 °C Then, to collect cells, 500 µL 2% FBS DMEM medium and 100 µL trypsin were added to each well. Furthermore, 500 µL of 2% FBS DMEM medium was added to the well to ensure complete cell collection. A mixture of 10 µL of LN229 and GBM8401 cells and 10 µL trypan blue in a 96-well plate was injected into the Neubauer chamber. The cells were count at the 4 corners of a Neubauer chamber with a microscope at 24, 48, 72, and 96 h after transfection. The previously described experimental procedure for the cell proliferation assay of each cell line was repeated 3 times. Finally, western-blot analysis was performed to detect cell cycle checkpoint expressions (cyclin B, cyclin E, CDK1, and CDK2), cell signaling pathways (ERK, phosphorylated-ERK, p38, and phosphorylated-p38), and cell matrix interaction (MMP2 and MMP9) on gliomas with siPLP2 and NC transfection.

### 4.8. Wound Healing Assay

First, 2 × 10^5^ LN229 and GBM8401 cells were seeded in a 12-well plate overnight. Then, glioma cells were scratched with 200 µL tips 48 h after transfection of NC or siPLP2. PBS was used to wash the cells, and the scratched area was photographed with a microscope at 0 h. A mixture of 1 mL DMEM medium and 2% FBS was slowly added into the well to maintain the cell growth. After a 16 h incubation at 37 °C, the medium was suctioned from the well and photographed with a microscope.

### 4.9. Cell Invasion Assay

Cells were seeded at 2 × 10^5^ cells/well in a 6-well plate for 24 h and transfected with NC or siPLP2. Trypsin was used to detach the transfected cells after 72 h. Then the cells were suspended in serum-free DMEM medium and adjusted to a density of 2 × 10^4^ cells. Transfected cells (500 µL) were added to the upper chamber and 10% FBS DMEM medium (500 µL) was added to the lower chamber. After a 24 h incubation, the membrane of the chamber was fixed with formalin, staining with crystal violet, and photographed with a microscope.

### 4.10. Soft Agar Colony Formation Assay

LN229 glioma cells with shPLP2 transfection were trypsinized and re-suspended in 1.5 mL 0.35% agarose and poured onto a 2 mL 0.5% agarose bed in 35 mm tissue culture dishes, then incubated overnight at 37 °C. After 3 weeks, all cultured cells were stained with 200 µL of crystal violet solution per well. After washing and drying, photographs of wells were taken using an imager and colonies were counted using image J software.

## 5. Conclusions

In conclusion, we indicated that PLP2 might have a “Yin-Yang” effect. PLP2 could prevent normal neuronal cell apoptosis and oxidative stress apoptosis, but unfortunately, PLP2 increases glioma aggressive behavior. Although the precise mechanism of PLP2 in gliomas remains unclear, PLP2 might be a potential target to prolong overall survival, delay disease progression, and suppress tumor infiltration.

## Figures and Tables

**Table 1:** The correlation of PLP2 immunostain score with WHO grades of gliomas.

	Average Intensity	Average % Tumor	Average Score	Correlation *
**Normal Brain Tissue**	0.55	10.95	10.95	
**Classification of Gliomas**				
Pilocytic astrocytoma	1	5	5	No correlation (*p* = 0.206)
Diffuse astrocytoma, IDH-mutant	0	0	0	
Diffuse astrocytoma, IDH-WT	1.31	35.38	59.23	
Anaplastic astrocytoma, IDH-mutant	0.67	6.67	6.67	
Anaplastic astrocytoma, IDH-WT	1.43	36.43	82.14	
Glioblastoma, IDH-mutant	1.2	33	46	
Glioblastoma, IDH-WT	1.53	41.32	79.41	
Oligodendroglioma, NOS	0.33	10	10	No correlation (*p* = 0.488)
Anaplastic oligodendroglioma, NOS	0.6	23	23	
Diffuse midline glioma, H3 K27M-mutant	1.47	44.67	87.33	
**WHO Grades of Gliomas**				
WHO grade I	1	5	5	Positive correlation (*p* = 0.039 *)
WHO grade II	1.06	28.82	47.06	
WHO grade III	1	26	47.33	
WHO grade IV	1.48	41.48	78.52	

* The correlation was analyzed by Pearson Product Method Correlation test.

**Table 2:** Univariate and multivariate analysis of risk factors associated with PLP2 expression.

Variable	Total	Univariate Analysis		Multivariate Analysis	
		OR (95% CI)	*p* Value	OR (95% CI)	*p* Value
**Sex**					
Male	47	1			
Female	29	1.18 (1.10–1.22)	0.507	1.28 (1.24–1.30)	0.279
**Age**					
<50	36	1			
≥50	40	2.38 (1.59–3.87)	0.011 ^#^	2.38 (1.59–3.87)	0.244
**IDH1 R132H**					
Negative	63	1			
Positive	13	0.46 (0.09–0.79)	0.065	0.46 (0.09–0.80)	0.069
**ATRX**					
Preserve	39	1			
Loss of expression	37	0.83 (0.76–0.86)	0.450	0.81 (0.74–0.85)	0.114
**H3K27M**					
Negative	67	1			
Positive	9	1.80 (1.44–2.00)	0.045 ^#^	1.68 (1.33–1.88)	0.029 *
**MGMT**					
Preserved	43	1			
Loss of expression	33	1.30 (1.17–1.63)	0.301	1.36 (1.22–1.71)	<0.001 *
**EGFR**					
Negative	69	1			
Positive	7	1.89 (1.42–2.16)	0.053	1.77 (1.31–2.03)	0.268
**EGFRvIII**					
Negative	58	1			
Positive	18	2.26 (2.14–2.49)	0.001 ^#^	2.29 (2.14–2.59)	0.013 *
**P53**					
Negative	36	1			
Overexpression	40	1.59 (1.39–2.14)	0.066	1.43 (1.27–1.87)	0.050
**Neurofilament**					
Negative	59	1			
Positive	17	1.17 (0.89–1.33)	0.582	1.16 (0.89–1.31)	0.091
**NF1**					
Negative	42	1			
Positive	34	1.29 (1.24–1.40)	0.302	1.18 (1.15–1.22)	0.002 *
**AxL**					
Negative	29	1			
Positive	47	2.21 (1.64–4.99)	0.006 ^#^	2.07 (1.50–4.67)	<0.001 *
**p-AxL**					
Negative	22	1			
Positive	54	0.97 (0.80–1.53)	0.923	0.97 (0.81–1.52)	0.004 *
**NUR77**					
Negative	27	1			
Positive	49	1.71 (1.35–3.10)	0.057	1.80 (1.42–3.28)	0.010 *
**H3Lys27**					
Preserved	67	1			
Loss of expression	9	1.59 (1.47–1.84)	0.021 ^#^	1.58 (1.43–1.80)	<0.001 *
**PDGFRA**					
Negative	5	1			
Positive	71	1.24 (0.41–1.76)	0.634	1.16 (0.30–1.66)	0.442

^#^*p* < 0.05, * *p* < 0.05.

**Table 3:** Multivariate Analysis for Overall Survival in human brain gliomas.

	Multivariate Analysis		
Variable	Hazard Ratio	95% Confidence Interval	*p* Value
Sex (male/female)	1.71	0.73–3.99	0.214
Age (<50/>50)	2.90	1.28–6.56	0.010 *
PLP2 (Negative/Positive)	1.01	0.99–1.03	0.030 *
IDH1 R132H (Negative/Positive)	2.69	0.87–8.30	0.086
ATRX (Preserve/Loss)	0.28	0.12–0.65	0.003 *
H3K27M (Negative/Positive)	2.20	0.56–8.76	0.262
MGMT (Unmethylated/ Methylated)	0.47	0.17–1.29	0.142
EGFR (Negative/Positive)	2.69	0.63–11.49	0.181
EGFRvIII (Negative/Positive)	0.90	0.39–2.06	0.805
P53 (Negative/Overexpression)	0.79	0.36–1.75	0.557
Neurofilament (Negative/Positive)	0.70	0.29–1.69	0.423
NF1 (Negative/Positive)	1.01	0.40–2.54	0.996
AxL (Negative/Positive)	4.03	1.32–12.27	0.014 *
p-AxL (Negative/Positive)	2.39	0.77–7.42	0.130
NUR77 (Negative/Positive)	0.35	0.14–0.85	0.021 *
H3Lys27 (Negative/Positive)	0.45	0.11–1.94	0.284
PDGFRA (Negative/Positive)	14.078	3.27–60.65	<0.001 *

* *p* < 0.05.
